# Comparative analysis of potentially inappropriate medication use in long-term care facility residents and community-dwelling elders: A matched cohort study

**DOI:** 10.1097/MD.0000000000031739

**Published:** 2022-12-09

**Authors:** Yumin Lee, Suhyun Jang, Hee-Jin Kang, Sunmee Jang

**Affiliations:** a College of Pharmacy and Gachon Institute of Pharmaceutical Sciences, Gachon University, Incheon, Republic of Korea.

**Keywords:** beers criteria 2019, long-term care facility, long-term care service, potentially inappropriate medication

## Abstract

As the population of the elderly in long-term care facilities has grown, the number of users of potentially inappropriate medication (PIM) is also increasing. With this study, we aimed to investigate the pattern of PIM usage and related factors among the elderly receiving long-term care services. Using the South Korean National Health Insurance Service Elderly Cohort Database, we conducted a retrospective matched cohort study. Elderly residents (n = 1980) in long-term care facilities in 2013 were selected and matched 1:1 with elderly persons living in the community applying propensity score method. The matching variables were sex, age, health insurance type, long-term care grade, Charlson’s Comorbidity Index score, presence of dementia, cerebrovascular disease, or Parkinson’s disease, and number of drugs prescribed. PIM use was assessed according to Beers criteria 2019. The prevalence of PIM was found to be higher among the elderly in long-term care facilities (86.77%) than among community-dwelling individuals (75.35%). Logistic regression showed that long-term care facility residents were 1.84 odds more likely to use PIM than community-dwelling older adults. We also confirmed that the average number of medications taken per day and the number of outpatient visits were the major influencing factors affecting PIM prescriptions. In addition, elders living in long-term care facilities were prescribed more PIM drugs acting on the central nervous system than community-dwelling older adults. The results of this study show that among those receiving long-term care services, older people in long-term care facilities use PIM more than do the elderly living at home. Medication management programs need to be developed to reduce the use of PIM in long-term care facilities.

## 1. Introduction

In South Korea, as the elderly population increases, the number of elderly people receiving long-term care services is also growing rapidly. Long-term care service is a social welfare system for the elderly who have difficulty performing daily activities independently, and services are provided at home or at a facility through a settled evaluation. According to a previous study, Korean long-term care service recipients show higher rates of disease, number of hospitalizations, and number of medications per prescription than the general elderly, but only the number of outpatient visits was lower.^[1]^ In addition, a study performed in Taiwan showed that the number of medications taken per day and the rate of emergency department visits were higher among the elderly who needed long-term care services than among the general elderly.^[2]^

Elderly residing in facilities have a higher hospital readmission rate^[3]^ than those living in the elderly dwelling-communities. Additionally, polypharmacy^[4]^ by the facility-dwelling elderlies leads to frequent drug-drug interactions.^[5]^ Meta-analyses of the prevalence of potentially inappropriate medication (PIM) among elderly in different residential types found the PIM prevalence rate to be 43%^[6]^ and 20.5%^[7]^ for long-term care facilities and elderly dwelling-communities, respectively, suggesting that the rate of PIM use among elderly residing in long-term care facilities was approximately 20% higher than that among those residing in communities. Therefore, it can be expected that among the elderly receiving long-term care, living in facilities increases the risk of PIM use.

In South Korea, the number of services received while dwelling in the community is about 2.4 times higher than that at long-term care facilities.^[1]^ Therefore, most studies have focused on community-dwelling older adults, and only a few studies have focused on the elderly living in long-term care facilities.^[8,9]^ Accordingly, there is a lack of research on the pattern of PIM use and the factors influencing it among elderly in facilities. Moreover, very few studies have compared the PIM use in elderly dwelling communities and long-term care facilities.

However, as the number of elderly in long-term care facilities is increasing,^[10]^ the rate of PIM prevalence is also increasing over time,^[6,11]^ and it is expected that the problems related to PIM among the elderly in facilities will increase. Therefore, this study identified whether there is a difference in the actual PIM prevalence, the difference in the types of PIM depending on the type of residence, and the factors affecting the PIM prevalence of the elderly living in a long-term care facility.

## 2. Methods

### 2.1. Data and study population

**Figure 1.**
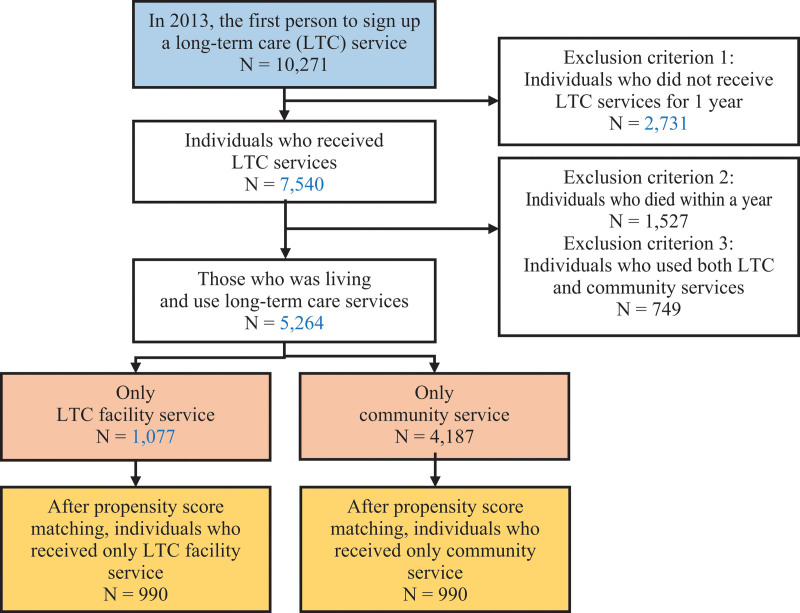
The flowchart shows the process of selecting the subjects. Among the 10,271 individuals who signed up for long-term care service, those who did not use long-term care service, those who died, and those who used service both in long-term care facility and community were excluded. As a result, 990 older adults in long-term care facility and 990 community-dwelling older adults were selected as final subjects.

In this study, cohort data of the elderly with national representativeness were generated by extracting information about 550,000 individuals, which constituted 10% of the 5.5 million people aged 60 years and over at the end of December 2002, as listed by the National Health Insurance Corporation. The National Health Insurance Service provides research data on insurance premium, medical use, long-term care service use, and drug use from 2002 to 2015 for elderly cohort subjects.^[12]^ In the elderly cohort data, the number of samples decreases annually as data on the elderly who have died are excluded every year. In this study, based on the most recent data from 2013, prescription patterns for 1 year were identified for the elderly receiving long-term care services for the first time. In 2013, 10,271 elderly individuals received long-term care ratings for the first time, and the elderly who did not receive long-term care services for 1 year and died were excluded from the study, resulting in a total of 5264 individuals. Among them, 1077 people used long-term care services at the long-term care facility within 1 year of their date of registration, 4187 people used long-term care services at home, and 749 people used the services at both places (Fig. 1).

### 2.2. Covariate and propensity score matching (PSM)

Among the elderly receiving long-term care services, data on community-dwelling older adults with the same characteristics as those living in long-term care facilities were extracted by matching propensity scores. Of the 1077 elderly in the facility, those at home were matched 1:1. As a result, approximately 92% matching was achieved, and 990 individuals from each facility and community were selected as the final study subjects. The matching variables were sex, age, health insurance type, long-term care grade, Charlson’s Comorbidity Index (CCI) score, presence of dementia, cerebrovascular disease, or Parkinson’s disease, and number of drugs prescribed. The types of health insurances were divided based on individuals who are insured by the National Health Insurance or recipients of medical benefits. Recipients of medical benefits, belonging to low-income class, pay fewer co-payments compared to national health insurance subscribers.^[13]^ The long-term care grade is determined by evaluating the physical and cognitive functions of the subject and ranges from grades 1 to 3, with grade 1 being the most dependent. To consider the severity of comorbid diseases, the CCI score was calculated based on the code of chronic disease diagnosed in the previous year from the first day of long-term care service (index date) and was divided into 0, 1, 2, and 3 points or more.^[14]^ The higher the score, the worse is the comorbidity. Dementia, cerebrovascular disease, and Parkinson’s disease were determined if the patient visited the hospital twice or more with the disease codes among the postmortem codes diagnosed in the year before the index date. For the number of prescribed drugs, only outpatient prescriptions were considered as the average number of drugs received per day in the previous year from the index date.

### 2.3. PIMs in older adults

PIMs were based on the Beers Criteria,^[15]^ the most recent of which is the 2019 version. Beers Criteria 2019 offers lists of PIMs in older adults, PIMs to avoid in older adults with certain conditions, medications to be used with considerable caution in older adults, medication combinations that may lead to harmful interactions, medications that should be avoided or dosed differently for those with poor renal function, and drugs with strong anticholinergic properties. This study used the first list, that is, PIMs. Medications not used in Korea were excluded from the list and those used only in Korea were included. A list of the PIMs used in this study is presented in Table S1, Supplemental Digital Content, http://links.lww.com/MD/H902. External drugs, for which the duration of administration cannot be easily considered, were excluded, and only oral and injectable drugs were included. The dosage and duration of each medication were excluded from the 2019 Beers Criteria.

### 2.4. PIMs prevalence/prescription

If at least 1 PIM-listed drug was included in the outpatient prescription for 1 year from the date of first receiving long-term care services, the patient was considered to have taken PIM (based on individuals). The PIM prevalence refers to the proportion of patients who undergo PIM. PIM prescription refers to the proportion of prescriptions that include PIM among prescriptions for 1 year.

### 2.5. Statistical analysis

After PSM, we determined whether each variable was well matched using the chi-squared test for categorical variables and the *t* test for continuous variables. Logistic regression was performed to determine the influence of the type of residence. In addition to the matched variables, medical use-related variables expected to influence PIM prescriptions (number of outpatient visits per year, number of medical institutions used, and type of medical institution frequently visited) were also included as variables. Another multivariate logistic regression analysis was performed to analyze the characteristics of PIM among older adults in long-term care facilities. In addition, we compared the list of PIMs that are frequently prescribed to the elderly residing in the service facilities and communities by prescription fraction. Data management and analysis were performed using SAS Enterprise 7.1.

### 2.6. Ethics statement

This study was reviewed and approved by the Institutional Review Board (IRB) of Gachon University, according to IRB regulations (IRB no: 1044396-202005-HR-100-01).

## 3. Results

A total of 990 elderly individuals living in long-term care facilities and 990 community-dwelling older adults were matched as the study subjects using PSM. There was a statistically significant difference in sex, age, health insurance type, long-term care level, CCI score, number of prescribed drugs, and presence of cerebrovascular and Parkinson’s diseases before matching, but no significant difference was observed after matching. After matching, there were about 3 times more females than males, and the elderly at level 3 formed the majority in the level of long-term care in both groups (Table 1). The average number of medications taken by elderly per day was 3.9 and 4.1 in long-term care facilities and communities, respectively. More than half of the elderly in both groups had at least one of the following diseases: dementia, cerebrovascular disease, or Parkinson’s disease.

**Table 1:** Characteristics of LTC*facility residents and community-dwelling elders.

Variables		Before matching†			After matching†		
		LTC* facility	Dwelling-community	*P* value	LTC facility	Dwelling-community	*P* value
Total number of participants		1077	4187		990	990	
Sex	Male	246 (22.8)	1117 (26.7)	.0101	233 (23.5)	299 (23.1)	.8734
	Female	831 (77.2)	3070 (73.3)		757 (76.5)	761 (76.9)	
Age		82.58	81.11	<.0001	82.34	82.35	.9438
National health security type	Health Insurance	875 (81.2)	3643 (87.0)	<.0001	814 (82.2)	813 (82.1)	.8931
	Medical aid	202 (18.8)	544 (13.0)		176 (17.8)	177 (17.9)	
Grade of long-term care	Grade 1	77 (7.2)	88 (2.2)	<.0001	59 (6.0)	60 (6.1)	.7300
	Grade 2	267 (24.8)	297 (7.2)		202 (20.4)	188 (19.0)	
	Grade 3	733 (68.1)	3715 (90.6)		729 (73.6)	742 (75.0)	
Charlson Comorbidity Index	0	295 (27.4)	1318 (31.5)	<.0001	284 (28.7)	287 (29.0)	.7768
	1	491 (45.6)	1525 (36.4)		433 (43.7)	450 (45.5)	
	2	186 (17.3)	724 (17.3)		168 (17.0)	155 (15.7)	
	≥3	105 (9.8)	620 (14.8)		105 (10.6)	98 (9.9)	
No. of pills per day		3.65	5.68	<.0001	3.91	4.06	.3835
Dementia or Cerebrovascular disease or Parkinson’s disease	Presence	656 (60.9)	1958 (47.8)	<.0001	584 (59.0)	564 (57.0)	.3870
	Absence	421 (39.1)	2229 (53.2)		406 (41.0)	426 (43.0)	
Dementia	Presence	461 (42.8)	1076 (25.7)	<.0001	402 (40.6)	412 (41.6)	.6810
	Absence	616 (57.2)	3111 (74.3)		588 (59.4)	578 (58.4)	
Cerebrovascular diseases	Presence	214 (19.9)	824 (19.7)	.8975	193 (19.5)	180 (18.2)	.4904
	Absence	863 (80.1)	3363 (80.3)		797 (80.5)	810 (81.8)	
Parkinson’s disease	Presence	41 (3.8)	266 (6.4)	.0013	40 (4.0)	37 (3.7)	.8163
	Absence	1036 (96.2)	3921 (93.7)		950 (96.0)	953 (96.3)	

LTC = long-term care.

† The LTC facility residents were matched with community-dwelling elders in a 1:1 ratio by propensity score method.

The average number of outpatient medical and medical institution visits per year in the elderly living in long-term care facilities group (Table 2) were 23.12 (standard deviation [SD] 17.22) and 3.27 (SD 1.94), respectively, while that for the community-dwelling group were 27.14 (SD 34.44) and 3.79 (SD 2.52), respectively. Compared to the elderly long-term care facilities, community-dwelling older adults most frequently visited clinics among all medical institutions, and the number of visits to a general hospital was similar in both groups.

**Table 2:** Medical utilization among LTC*facility residents and community-dwelling elders.

Variables		LTC facility	Dwelling-community	*P* value
No. of outpatient visits per person	Average (SD)	23.12 (17.22)	27.14 (33.44)	.0008
	<10	13.13%	27.37%	
	10–20	34.14%	27.37%	
	20–30	29.09%	18.08%	
	>30	23.64%	27.17%	
No. of outpatient institutions per person	Average (SD)	3.27 (1.94)	3.79 (2.52)	<.0001
	<3	40.81%	35.96%	
	3–4	37.68%	34.85%	
	>4	21.52%	29.19%	
Type of predominant medical center	General Hospital	32.22%	34.95%	<.0001
	Hospital	34.44%	15.15%	
	Clinic	33.33%	49.90%	

LTC = long-term care, SD = standard deviation.

The PIM prevalence was 86.77% and 75.35% in the elderly residing in facilities and communities, respectively (*P* < .0001, Table 3). Based on the prescription measure, 46.28% and 36.08% of all prescriptions for the elderly in facilities and in communities included PIM, respectively. When comparing prescriptions that included PIM, the average number of PIMs was 1.52 for the elderly in facilities but 1.30 for those at home.

**Table 3:** Differences in PIM use between LTC*facility residents and community-dwelling elders.

Variables	LTC facility (N = 990)	Dwelling-community (N = 990)	*P* value
PIM Prevalence (per old adult)	86.77% (859)	75.35% 746	<.0001
PIM Prevalence (per prescription)	46.28%	36.08%	<.0001
Average number of PIMs* (per prescription)	1.52	1.30	<.0001
Odds ratio*	1.843 (95% CI: 1.42–2.39)	ref	<.0001

CI = confidence interval, LTC = long-term care, PIM = potentially inappropriate medication.

* Logistic regression analysis was conducted using the following variables: number of outpatient visits per person, number of outpatient institutions per person, and type of predominant medical center, among the elderly in LTC facilities or dwelling-communities after propensity score matching.

**Figure 2.**
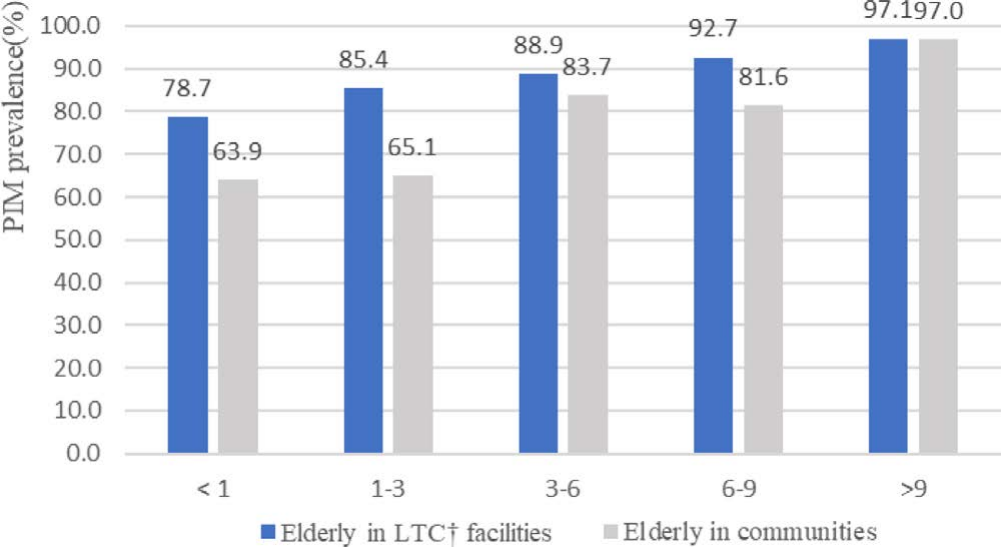
PIM prescription rate for the elderly in the long-term facility was higher than that for community-dwelling older adults regardless of the number of medications. The lower the number of medications taken per day, the greater the difference in PIM prescription rate between the elderly in long-term care facilities and communities. PIM = potentially inappropriate medication.

The PIM prescription rate for the elderly in the facility was higher than that for community-dwelling older adults in all areas, regardless of the number of medications (Fig. 2). In addition, as the number of prescription drugs increases, the PIM prescription rate also increases. It was observed that the number of PIM prescriptions received by elderly in facilities was higher by an odds ratio (OR) of approximately 1.84 compared to that received by the elderly at home (95% confidence interval [CI] = 1.42–2.39).

Another logistic regression analysis was performed to identify factors affecting PIM prescriptions for the elderly in a facility. Table 4 shows that the average number of medications taken per day and the number of outpatient visits were the major influencing factors. For the group with an average number of medications taken as <3 per day, as the number of medications increased, the likelihood of prescribing PIMs increased (3 to <5: OR = 1.48, 95% CI = 0.849–2.581; 5 to <10: OR = 2.491, 95% CI = 1.391–4.461; >10: OR = 3.407, 95% CI = 0.966–12.023). The likelihood of prescribing PIMs also increased as the number of outpatient visits increased (10 to <20: OR = 2.935, 95% CI = 1.747–4.931; 20 to <30: OR = 4.296, 95% CI = 2.368–7.792; >30: OR = 8.396, 95% CI = 3.931–17.933; reference group: <10).

**Table 4:** Predictors of PIM use among elderly in LTC facility.

		Odds ratio	95% CI	*P* value
Sex	Male	ref		.3824
	Female	0.796	0.478–1.328	
Age*	71–74	ref		.5190
	75–79	1.193	0.539–2.639	
	80–84	1.124	0.525–2.408	
	85–89	0.746	0.347–1.602	
	>90	1.091	0.467–2.552	
National health security type	Health Insurance	ref		.7448
	Medical aid	0.913	0.529–1.576	
Grade of Long-term care	Grade 1	0.897	0.382–2.106	.4789
	Grade 2	0.735	0.446–1.209	
	Grade 3	ref		
Charlson Comorbidity Index	0	ref		.0779
	1	2.299	1.199–4.409	
	2	1.595	0.716–3.556	
	≥3	1.62	0.644–4.076	
No. of pills per day	<3	ref		.0078
	3–5	1.48	0.849–2.581	
	5–10	2.491	1.391–4.461	
	>10	3.407	0.966–12.023	
Dementia†		0.906	0.500–1.642	.7448
Cerebrovascular diseases†		0.565	0.302–1.058	.0744
Parkinson’s disease†		1.82	0.496–6.679	.3665
No. outpatient visits per person	<10	ref		<.0001
	10–20	2.935	1.747–4.931	
	20–30	4.296	2.368–7.792	
	>30	8.396	3.931–17.933	
No. outpatient institutions per person	<3	ref		.0805
	3–4	1.429	0.860–2.374	
	>4	0.808	0.496–1.317	
Type of predominant medical center	General Hospital	ref		.1318
	Hospital	1.181	0.715–1.953	
	Clinic	1.86	0.987–3.506	

CI = confidence interval, LTC = long-term care, PIM = potentially inappropriate medication.

* Continuous variable.

† Reference: absence of the disease.

The most frequently prescribed PIM ingredient for the elderly living in long-term care facilities is quetiapine, which accounts for approximately 15% of the all PIM prescriptions, followed by alprazolam (7.61%), diazepam (7.36%), zolpidem (7.02%), and chlorpheniramine maleate (6.00%). In contrast, in the case of the matched community-dwelling elderly population, the most frequent PIMs were diazepam (13.22%), chlorpheniramine maleate (9.31%), amitriptyline (6.28%), quetiapine (5.77%), and alprazolam (5.53%) (Table 5).

**Table 5:** Top 15 prescribed drugs in PIM.

LTC facility		Dwelling-community	
Ingredient	Ratio	Ingredient	Ratio
Quetiapine	14.64	Diazepam	13.22
Alprazolam	7.61	Chlorpheniramine Maleate	9.31
Diazepam	7.36	Amitriptyline	6.28
Zolpidem	7.02	Quetiapine	5.77
Chlorpheniramine Maleate	6.00	Alprazolam	5.53
Amitriptyline	5.77	Dimenhydrinate	4.60
Lorazepam	4.81	Hydroxyzine HCl	4.40
Risperidone	4.62	Zolpidem	4.12
Glimepiride	4.20	Lorazepam	3.40
Hydroxyzine HCl	4.10	Metoclopramide	3.18
Dimenhydrinate	2.77	Diclofenac	3.14
Meloxicam	2.12	Meloxicam	2.56
Clonazepam	2.06	Rabeprazole Sodium	1.92
Triazolam	2.05	Digoxin	1.75
Digoxin	2.03	Risperidone	1.70

LTC = long-term care, PIM = potentially inappropriate medication.

## 4. Discussion

It was found that approximately 80% of the elderly residing in both facilities and communities were prescribed PIMs during the year (86.77% at facilities vs 75.35% in communities). This value is similar to or higher than 80.96%,^[16]^ obtained using the 2009 to 2011 health insurance outpatient claim data for the elderly in Korea, 70.3%, PIM use rate of all seniors obtained using the 2011 health insurance claim data,^[17]^ and 73.0%, obtained using health insurance data of 2016.^[18]^

In Quebec, Canada, the PIM prevalence was 48.3% of the total elderly,^[19]^ which was 42.7% in New Zealand^[20]^ and 44.2% in Brazil.^[21]^ These value are very low compared to the values from South Korea. In contrast, the PIM prescription rate was 87.3% in India,^[9]^ 82.6% in 2011^[22]^ and 86.22% in 2015 in Taiwan,^[23]^ and 63.98% in Thailand,^[24]^ indicating that Asia has a higher PIM usage than the United States of America and Europe. Chang et al^[23]^ suggested that the reason for the high PIM prescription rate in Asian countries is that the number of visits to medical institutions and the rate of polypharmacy are higher in Asia than those in other continents. In fact, in South Korea, the number of outpatient visits per year is 17.2 (OECD average of 6.8), and the polypharmacy rate is 70.2% (OECD average of 48.3%), all of which are the highest among OECD countries. In particular, the average number of outpatient visits for the elderly in South Korea is 35 per year.^[1]^ This high number of outpatient visits can be explained as one of the factors resulting in higher prevalence of PIM in South Korea than that in other countries. In addition, there is a lack of any comprehensive drug management service provided by physicians or pharmacists that can review the patient’s overall prescription. In the case of PIM-related studies, the results may vary as the study design, sociodemographic and clinical status of the elderly, and drugs usage differ. Nevertheless, a combination of these factors may have led to the higher prevalence of PIM in South Korea than that in other countries.

The probability of taking PIM (OR of 1.83) was higher for the elderly living in the long-term care facilities than for those dwelling in communities. PIM prevalence and prescription also showed the similar trend. This result is similar to that of previous studies. In a 2017 study conducted in France that used the same PIM standards as used in this study, the PIM prevalence among the elderly in long-term care facilities was 71%^[25]^ but that among the total proportion of the elderly was 46.7%,^[26]^ indicating that the elderly in facilities was high. In Ontario, Canada, older adults in facilities showed lower PIM use than those dwelling in communities. The findings of this study differed from those of others because in Ontario, the clinical pharmacist drug management service in long-term care facilities is compulsory.^[27]^ The average number of medications taken per day is one of the factors influencing PIM prescriptions, as has been reported in many studies.^[6,16,19,28,29]^ In cases where the average number of medications taken per day was low, the difference in PIM prescription rates between community-dwelling older adults and the elderly in a facility was large. In particular, as shown in Figure 2, a higher number of medications taken per day leads to a higher PIM prevalence rate. It was found that the higher the frequency of visits to medical institutions, the higher was the PIM use rate, which is consistent with the findings of a previous study.^[30]^ Although not statistically significant in this study, the use of PIM was higher in grade 3 than in grade 1 individuals, which makes it difficult for elderly to lead an independent life in the long-term care grade (OR = 1.15, *P* = .4789). Another study on PIM usage in Korean long-term care facilities also showed a similar result with the following CCI scores: CCI point 1, OR = 2.299 (1.199–4.409); point 2, OR = 1.595 (0.716–3.556); >point 3, OR = 1.62 (0.644–4.076); reference group: point 0, *P* = .0779.^[31]^ In addition, the result that PIM prescription decreased in the presence of cerebrovascular disease or dementia (dementia: OR = 0.906 (0.500–1.642), *P* = .7448; cerebrovascular disease: OR = 0.565 (0.302–1.058), *P* = .0744) is consistent with that of previous studies, indicating that PIM prevalence decreases with cognitive impairment.^[6,32]^ The reason underlying this trend is hypothesized to be the carefulness among doctors while prescribing PIMs due to the serious condition of the elderly.

The ingredients of PIM mainly used in long-term care facilities and communities were found to be different. In the case of the elderly residing in facilities, 9 out of the top 15 most frequent PIMs were antipsychotic drugs (quetiapine, alprazolam, diazepam, zolpidem, amitriptyline, lorazepam, risperidone, clonazepam, and triazolam), which accounted for approximately 56% (55.94) of all prescription PIMs. In contrast, in the case of matched community-dwelling older adults, 7 of the top 15 ingredients were psychotropic drugs (diazepam, amitriptyline, quetiapine, alprazolam, zolpidem, lorazepam, and risperidone), accounting for 40% (40.02) of all prescription PIMs, which is consistent with the findings of previous studies in South Korea^[31,33]^ and other countries. For community-dwelling older adults, benzodiazepines, pain medications, metoclopramide, and antidepressants were suggested as frequent PIM drugs in Canada, Quebec, India, and Brazil.^[8,9,21]^ The meta-analysis revealed that the most frequent PIM ingredients for the elderly living in long-term care facilities were long-acting benzodiazepines, fluoxetine, tricyclic antidepressants, anticholinergics, anti-inflammatory analgesics, digoxin, and proton pump inhibitors, in that order. Compared to that in communities, ingredients acting on the central nervous system were prescribed more in facilities.^[6]^ Subsequently, it is necessary to evaluate the factors and appropriateness that can explain the differences in antipsychotic drug prescription patterns between these 2 groups. In particular, it is necessary to prioritize the evaluation of prescription patterns of antipsychotic drugs for the elderly living in long-term care facilities.

This study had several limitations. The first is the definition of a PIM. In this study, the Beers 2019 Criteria was used as a standard, and as this has been defined in the United States, there may be clinical differences in its application to the elderly in Korea. In addition, there are cases in which PIMs are appropriately used depending on the clinical condition of the patient. However, as the data used in this study did not reveal the reason for the prescription, it was not possible to consider whether PIM would be inappropriate for the patient in actual clinical situations. Even for drugs on the PIM list, the dosage conditions suggested by the Beers Criteria were not considered. Second, there were limitations in the data. As this study used cohort data established for the elderly aged 60 years and older in 2002, all subjects in the 2013 study (the base year of the study) were 71 years and older. As the standard for the elderly is generally 65 years or older, this study did not include data from individuals aged 65 to 70 years. In addition, there are cases in which the diagnosis in the health insurance claim data does not reflect actual clinical symptoms. Even if there was a prescription history for the drug, it was not possible to confirm whether the patient actually took the prescribed drug. In addition, as the health insurance claims data contain only records of drugs that are covered by insurance, there is no the usage of non-covered and over-the-counter drugs. Antihistamines, antispasmodics, muscle relaxants, and anti-inflammatory analgesics are taken as over-the-counter medicines by many older people, but these were not considered in this study.^[20]^ Major geriatric diseases, such as dementia, cerebrovascular disease, and Parkinson’s disease, were thought to influence PIM prescription, but the severity of the diseases could not be considered. Moreover, 40% of the elderly receiving long-term care services whether they live in facilities or at home have dementia. The caregiver may be a factor affecting PIM use in patients with dementia. Unlike the situation at home, long-term care facilities have professional caregivers, and this difference may influence the prevalence of PIM. However, due to limitations in the data, we did not consider the characteristics of caregivers. Therefore, the results should be interpreted with caution, and further studies of PIM are needed, in which the characteristics of the caregivers are included.

The strengths of this study are as follows: This study suggests that PIM prevalence could increase by living in long-term care facilities, even for elderly with similar conditions. It also identifies that the frequently used PIMs can vary according to the type of residence, even for elderly with similar conditions. Therefore, in order to solve the problems caused by PIM usage in the facilities, it is necessary to adopt a different approach than that adopted for the elderly communities. Considering that the drug management program in long-term care facilities shows that the use of PIM in elderly patients at long-term care facilities was lower than that in community-dwelling older adults, it seems that it is essential to conduct medication management programs in long-term care facilities. Examples of current medication management programs are Medication Therapy Management,^[34,35]^ Drug Regime Review in the United States^[36,37]^ and Residential Medication Management Review in Australia.^[38,39]^ Likewise, in Korea, a program should be developed in which pharmacists visit long-term care facilities regularly to comprehensively review the medications. This study can be used as a basis for policy development and research related to PIM management in long-term facilities.

## 5. Conclusions

This study confirmed that among the elderly receiving long-term care services, those at long-term care facilities were more likely to be prescribed PIM than community-dwelling older adults. In addition, older adults living in long-term care facilities were prescribed more PIMs acting on the central nervous system than community-dwelling older adults, indicating that PIM prevalence and prescription patterns differed depending on the type of residence.

## Acknowledgments

We would like to thank Editage (www.editage.co.kr) for English language editing.

## Author contributions

YL and SJ conceived and designed the study. YL collected and analyzed the data and wrote the manuscript. SJ, KH, and SJ participated in drafting the article or critically revising it for content.

**Conceptualization:** Yumin Lee, Sunmee Jang.

**Data curation:** Yumin Lee, Suhyun Jang, Heejin Kang.

**Formal analysis:** Yumin Lee, Suhyun Jang, Heejin Kang.

**Project administration:** Yumin Lee.

**Supervision:** Sunmee Jang.

**Writing – original draft:** Yumin Lee.

**Writing – review & editing:** Sunmee Jang.

## Supplementary Material


