# Supplementary material for: Comparative analysis of potentially inappropriate medication use in long-term care facility residents and community-dwelling elders: A matched cohort study
Source: Medicine (Baltimore). 2022 Dec 9;101(49):e31739. doi: 10.1097/MD.0000000000031739 (PMC9750672; doi:10.1097/MD.0000000000031739)
Supplement: Supplementary file 1 [file medi-101-e31739-s001.pdf]

Supplemental Material

Table S1. Beers Criteria for Potentially Inappropriate Medication Use in Older Adults, Modified According to the Korean Context

Organ System	Therapeutic Category	Drugs
**Anticholinergics**	First-generation antihistamines	Brompheniramine
		Carbinoxamine
		Chlorpheniramine
		Clemastine
		Cyproheptadine
		Dexbrompheniramine
		Dexchlorpheniramine
		Dimenhydrinate
		Diphenhydramine (oral)
		Doxylamine
		Hydroxyzine
		Meclizine
		Promethazine
		ebastine
		oxatomide
		Triprolidine
	Antiparkinsonian agents	Benztropine (oral)
		Trihexyphenidyl
	Antispasmodics	Atropine (excludes ophthalmic)
		Belladonna alkaloids
		Clidinium-Chlordiazepoxide
		Dicyclomine
		Hyoscyamine
		Propantheline
		Scopolamine (hyoscine)
**Antithrombotics**	Dipyridamole, oral short-acting (does not apply to the extended-release combination with aspirin)	Dipyridamole
	Ticlopidine	Ticlopidine

**Anti-infective**	Nitrofurantoin	Nitrofurantoin
**Cardiovascular**	Peripheral alpha-1 blockers	Doxazosin
		Prazosin
		Terazosin
	Central alpha blockers	Clonidine
		Guanabenz
		Guanfacine
		Methyldopa
		Reserpine (>0.1 mg/d)
	Disopyramide	Disopyramide
	Dronedarone	Dronedarone
	Digoxin	Digoxin
	Nifedipine, immediate release	Nifedipine, immediate release
	Amiodarone	Amiodarone
**Central nervous system**	Antidepressants, alone or in combination	Amitriptyline
		Amoxapine
		Clomipramine
		Desipramine
		Doxepin >6 mg/d
		Imipramine
		Nortriptyline
		Paroxetine
		Protriptyline
		Trimipramine
	Antipsychotics, first- (conventional) and second- (atypical) generation	prochlorperazine
	(1st generation)	chlorpromazine
		promazine
		triflupromazine
		levomepromazine
		fluphenazine
		trifluoperazine
		perphenazine
		thioridazine
		mesoridazine
		pericyazine
		pipotiazine

	pimozide
	fluspirilene
	molindone
	clotiapine
	loxapine
	haloperidol
	bromperidol
	benperidol
	droperidol
	thiothixene
	clopenthixol
	zuclopenthixol
	fluphenthixol
	olanzapine
(2nd generation)	clozapine
	brexipiprazole
	aripiprazole
	asenapine
	quetiapine
	lurasidone
	paliperidone
	risperidone
	iloperidone
	ziprasidone
	pimavanserin
Barbiturates	Amobarbital
	Butabarbital
	Butalbital
	Mephobarbital
	Pentobarbital
	Phenobarbital
	Secobarbital
Benzodiazepines (short, intermediate)	Alprazolam
	Estazolam
	Lorazepam
	Oxazepam
	Temazepam
	Triazolam

		etizolam
		Midazolam
		brotizolam
		clotiazepam
	Benzodiazepines (long)	Clorazepate
		Chlordiazepoxide (alone or in combination with amitriptyline or clidinium)
		Clonazepam
		Diazepam
		Flurazepam
		bromazepam
		flunitrazepam
		Flutoprazepam
		Pinazepam
		Nordazepam
		Quazepam
	Nonbenzodiazepine, benzodiazepine receptor agonist hypnotics	Eszopiclone
		Zolpidem
		Zaleplon
	Ergoloid mesylates (dehydrogenated ergot alkaloids)	Ergoloid mesylates (dehydrogenated ergot alkaloids)
	Isoxsuprine	Isoxsuprine
**Endocrine**	Androgens	Methyltestosterone
		Testosterone
	Estrogens with or without progestins	Estrogens with or without progestins
	Megestrol	Megestrol
	Sulfonylureas, long-duration	Chlorpropamide
		Glyburide (Glibenclamide)
**Gastrointestinal**	Metoclopramide	Metoclopramide
	Proton-pump inhibitors	omeprazole
		esomeprazole
		lansoprazole
		dexlansoprazole
		rabeprazole
		pantoprazole
		ilaprazole

**Pain medications**	Meperidine	Meperidine(pethidine)
	NSAID	Aspirin >325 mg/d Diclofenac
		Diflunisal
		Etodolac
		Fenoprofen
		Ibuprofen
		Ketoprofen
		Meclofenamate
		Mefenamic acid
		Meloxicam
		Nabumetone
		Naproxen
		Oxaprozin
		Piroxicam
		Sulindac
		Tolmetin
		Indomethacin
		Ketorolac, includes parenteral
	Pentazocine	Pentazocine
	Skeletal muscle relaxants	Carisoprodol
		Chlorzoxazone
		Cyclobenzaprine
		Metaxalone
		Methocarbamol
		Orphenadrine
	Genitourinary	Desmopressin
